# Progress in Medicine

**Published:** 1896-12-19

**Authors:** 


### Progress in Medicine.

### DISEASES OF THE NERVOUS SYSTEM.

*(Continued from page 183.)*

**Insanity.**—The question of the domestic treatment of the insane has received some attention recently, a question always difficult to form a reliable opinion on, seeing that as a rule alienists have experience of the asylum methods alone, and thus the only practitioners who can speak from experience are naturally, however unintentionally, partial in their conclusion. But Connolly Norman,[21] the resident medical superintendent of the Richmond (Dublin District) Asylum, in his two most recent addresses to his section of State medicine of the Royal Academy of Medicine in Ireland, discusses the question in a most impartial manner. In his first address he drew attention to some of the disadvantages resulting from the treatment of the insane in asylums. He remarked that these are not very likely to be exaggerated by one who has been twenty years a asylum officer, and who cannot pretend to have entirely raised himself above the *idola specûs*. They are, however, considerable. In the large asylums individual treatment—the one thing likely to benefit our patients—becomes almost impossibles. Besides, all patients do not require the discipline of an asylum, and where not needed this discipline and the routine of an institution are trying. In his opinion, they are distinctly injurious as well. Here, as in so many other ætiological questions, it is not always easy to sepaiate cause and effect. The inertia, the indolence, the self-absorption, the lack of all human interest in life, which are the characteristics of dementia, are most distinctly fostered by the unhealthy surroundings of asylum society. He has no doubt whatever that many curable cases, when the acute stage is over, are immensely benefited by a change of surroundings. The danger just then is that of allowing the patient to yield to the apathetic tendency which often exists after the defervescence of the acute symptoms. This is best met by restoring him to a life in which he can take an interest. There is a great danger lest, in the dull atmosphere of an asylum, he may grow more and more indolent and timid. He readily falls into a monotonous groove, from which he becomes too apathetic to strive to emerge. Meanwhile the period during which cure is probable passes away, and our patient figures as a chronic dement. Even for the chronic dement for whom there is no cure the asylum in many cases has disadvantages, though in no wise is it to be held that all incurable cases of insanity, all chronic cases, or all cases even of chronic and hopeless dementia are fit to be at large, and should not be retained in asylums. But a large number do not require the special treatment of an asylum. Norman refers to the advantage of some system of boarding out suitable cases as has been done at Gheel for many generations past. He himself has been converted from the opinion he expressed five years ago that the management of the insane has become, so to speak, an hereditary tact with the inhabitants of the place, and that this could no more be exported than the other traditions of Gheel. Gheel, in fact, has been imitated, and imitated with success, and it turns out that the only great difficulty in imitating Gheel is the difficulty of getting people to try. It was determined to establish, on the lines of Gheel, a colony in the Walloon country and a district in the Belgian Ardennes called Lieurneux was selected. It is thinly populated, and it was designed to plant lunatics to the number of 1,000 in the houses of the 2,500 inhabitants. Lieurneux was selected because it was remote, because it was not traversed by railways or intersected by canals, and because it was supposed that the inhabitants, being poor and without any special industry, supporting themselves chiefly by agriculture, would be glad to accept lunatics as boarders. So they proved eventually, though loudly protesting when the proposal was first made. There are now several hundred patients in the district, and all classes of the insane are admissible except the suicidal, homicidal, incendiaries, those who are apt to offend against public decency, and those in whom it is necessary to employ "continuous mechanical restraint," the last being a class which we do not find to exist in this country. The patients live in the houses of the hosts, mixing freely with them in domestic life, and, in the case of public patients, following, as far as they are able, the trade or calling of the hosts. For the public patients the State pays ninety centimes per diem in the case of patients who do work at a trade or on the farms, and one franc per day for those who do not work. The reception of lunatics has become popular among the sane population, and early prejudices were soon overcome. During the earlier period Lieurneux had, among other difficulties, to contend with the circumstance that there was no proper central hospital, but there is now a well fitted dispensary. In spite of this and many other disadvantages and discouragements which befel the young colony, the results have been amazingly good, especially when one considers what Dr. Deperon points out in his report, that the patients sent to Lieurneux from the closed asylums have been of the worst classes, "most unhappy beings reduced to vegetative life, idiots on the lowest round of the ladder, paralytics in the last stage, and the like—patients whom up to the present we have felt bound to admit in our wish to people our young colony."

The French Government also has now established a colony for senile dements at the country town of Dun-le-Roi, not far from Bourges; it is a quiet little town that has seen better days, with a population of 4,200. During the first year and a-half of its existence 143 patients were received. The capitation cost per diem was 1 franc 94 c., considerably less than the grant in the asylums of the Department of the Seine. The Scotch boarding-out method of dealing with harmless lunatics differs from that of Gheel, Lieurneux, and Dun-le-Roi, inasmuch as there is no distinct colony, and patients are settled wherever it happens to be convenient to place them. Norman refers to the somewhat similar system started recently in Berlin, and states that he believes that it has several points of superiority over the Scotch plan. The patients are not confined to any one special area; they are scattered all through Berlin and its suburbs. They are boarded, some with relatives, some with strangers, when their own relatives have refused to take them, and have protested against their discharge. The patients are visited by a medical officer of the asylum at least once every month, this being a much better plan, in Norman's opinion, than the system in use in Scotland, where the unhappy lunatic who is boarded out is visited by no one who can claim to have any particular knowledge of the insane, except the Assistant Commissioner of Lunancy, who visits him once a year.

Turning now to another equally interesting communication on the subject of non-asylum treatment of the insane by W. F. Robinson,[22] of Albany, he commences by saying that while some claim that the asylum itself has some wonderful curative power, and therefore every case should be at once hurried to an asylum, certain authorities, notably Weir Mitchell, of Philadelphia, declare that this is a mere supposition, and that cases are very much better treated outside of asylums than in. Neither of these statements, representing opposite extremes, are true. One thing, he says, may be put down as true, with very few exceptions, and that is that the worst treatment of all for this class of cases is home treatment. The treatment in an institution, however poor, can hardly be worse for the patient than the treatment at home, and the advocates of the asylum are therefore right in urging immediate commitment to an asylum, when this is the only alternative. In the vast majority of cases of insanity there are only these two alternatives, so that the case is practically settled. To people of wealth there is another course open—the small private asylum. Patients in these private retreats undoubtedly get most excellent care, but there is very little evidence that any very earnest effort is made to cure the cases. For certain cases there is, in the author's opinion, a form of treatment combining all the undoubted advantages of the asylum without any of its drawbacks. This method cannot be applied except in certain selected cases, a question which must be settled by a personal examination by the expert. Where it can be applied and all the requirements can be fulfilled, it certainly offers the very best chance for recovery. It requires, first and foremost, removal from home, which is an absolute necessity, the services of a specialist in mental diseases, and also those of a trained nurse; a method hard to name, but, for want of anything better, Robinson terms it the sanitarium treatment. The author reminds us that the insane are often extremely sensitive to unkind or rude treatment; and, on the other hand, they are quite grateful for favours or kindnesses. Some of these unfortunates get the reputation for being violent, when the fault lies with those in whose care they are. As regards mental treatment, the keynote of this treatment is to treat the patient as if he or she were sane—in other words, in one's intercourse with them to ignore the whole insane fabric entirely. He insists on the importance of cheerful surroundings, and every endeavour should be made to provide interests for the patients.

**Treatment by Thyroid Extract.**—Brush[4] reports six cases of mental disturbance treated with thyroid extract: (1) Woman, age fifty-one, five years insane, delusions of doubt and fear; mental condition remained in many respects as impaired as before treatment by thyroid, but general condition improved, and delusions less in evidence. (2) Chronic delusional insanity, violent, untidy, destructive, for one year previous to treatment; had been looked upon as hopeless, but she improved immensely under thyroid treatment, and was able to be removed to her home, where she lived in quiet and comfort. (3) Child, case of simple melancholia of several months' duration. Patient became convalescent. (4) Woman with attacks of recurrent maniacal excitement. The last attack had been very prolonged and very violent; and when the patient was put upon the thyroid treatment she was noisy, turbulent, destructive, and untidy. Within one month the patient was among convalescent patients—quiet, lady-like, but somewhat depressed. She continued to improve and became practically cured. Nos. (5) and (6) were cases of chronic melancholia in men in which no improvement was manifest, the first four cases being females. Brush stated that he was inclined to endorse the views of Dr. Bruce, as recently expressed in his journal of mental science, that the thyroid undoubtedly produced a more or less feverish condition, the action and reaction to which is of considerable benefit to the patient, and that it is a direct cerebral stimulant. In the discussion following this paper Francis Dercum[23] stated that in the first place it seemed to him that a drug which increases blood pressure, which increases vascular tension, which increases the pulse-rate, and is a powerful febrifacient, is a drug which very likely will modify chronic pathological changes, especially slight changes such as those upon which insanity often depends. The drug also may act as a stimulant, and indirectly, by favouring the excretion of various toxic substances upon which the insanities probably depend. A. R. Moulton[24] remarked that when he noted the wonderful changes that have been reported from the use of thyroid extract in insanity, he could but remember the fact that in many forms of insanity, if one does anything unusual the patient will improve for a time, and when Dr. Brush spoke of his case of disorderly mania which became pleasant and agreeable under the use of thyroid extract, he was reminded of an old gentleman who insisted upon spitting on his doctor and using him very roughly, but who got into a much better state under the use of tobacco. He was loth to give much credence to some of the statements of the wonderful results following the use of this remedy, when he knew that very simple means will bring about decided changes in mental disease. At the same time he considered that such investigations as Dr. Brush had reported were of the greatest value. William Osler[25] stated that whatever may be the function of the internal secretion of the thyroid, one very important work that it has to do is to stimulate brain metabolism. The change in the physical condition of myxœdematous patients under his exhibition of thyroid gland has not been so striking, in his experience, as the change in the mental condition. The patients have become bright and intelligent, and have been able to resume their social duties. We must regard the thyroid extract as containing a most potent cerebral stimulant which does alter in some way the metabolism of the nerve centres and stimulates them in a most extraordinary manner. Perry[26], as the result of his studies of the blood in thyroid feeding in insanity, concludes that in some forms of insanity there is a sluggish action of some of the tissues intimately connected with the function of hæmatosis, which tissues, being stimulated by a vigorous course of thyroid, elaborate and turn into the circulation some principle which has a beneficial action on the cerebral cortex.

[20] Dublin Journ. of Med. Sc., Feb., 1896. [21] Amer. Journ. of Insanity, April, 1896. [22] Journ. of Nervous and Mental Diseases, Vol. XXI., No. 4. [23] Ibid. [24] Ibid. [25] Ibid. [26] Med. Rec., Aug. 29, 1896.

